# Supplementary figures and images for: Lead ethyl dithiocarbamates: efficient single-source precursors to PbS nanocubes
Source: R Soc Open Sci. 2019 Oct 30;6(10):190943. doi: 10.1098/rsos.190943 (PMC6837187; doi:10.1098/rsos.190943)

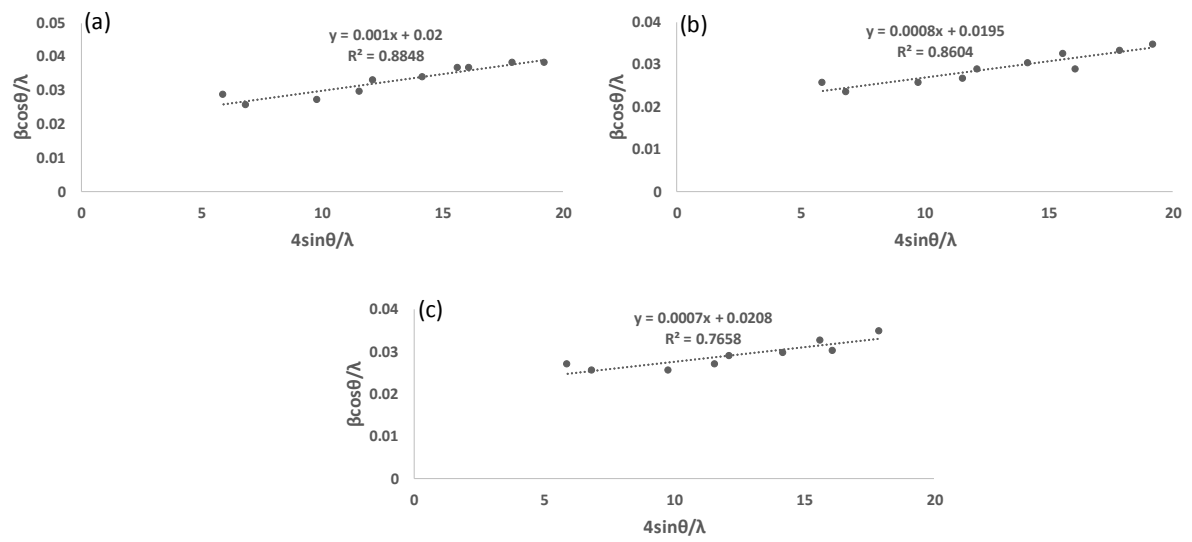

Figure 1: Williamson-Hall plots of PbS at (a) 250, 300, 400 °C

Supplement: Williamson Hall plots [file rsos190943supp1.pdf]

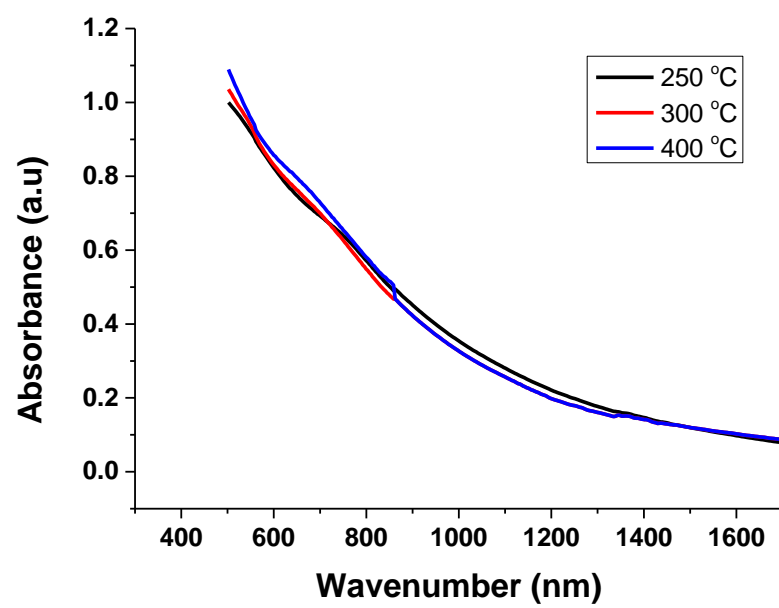

Figure 2: Uv-vis-nir of PbS at 250, 300, 400 °C

Supplement: Uv-vis-nir plots [file rsos190943supp2.pdf]
